# A pharmacist-led framework for antibiotic-associated liver injury: from pharmacovigilance signals to bedside surveillance with the updated RUCAM and susceptibility markers

**DOI:** 10.3389/fphar.2026.1956601

**Published:** 2026-09-22

**Authors:** Chao Liu, Dan Luo, Ruifeng Lin

**Affiliations:** Zigong First People’s Hospital, Zigong, China

**Keywords:** antibiotics, causality assessment, clinical pharmacist, drug-induced liver injury, pharmacovigilance, updated RUCAM

## Abstract

Antibiotics account for more cases of idiosyncratic drug-induced liver injury (DILI) than any other drug class. At the bedside, attributing injury to a particular antibiotic is difficult: there is no confirmatory test, and latency and biochemical pattern vary markedly among agents. Spontaneous-reporting databases add a second kind of evidence. Disproportionality analyses repeatedly identify strong reporting signals for amoxicillin-clavulanate and nitrofurantoin, but these describe reporting patterns rather than incidence, rest on reports that are seldom assessed by a validated causality method, and cannot establish causation in an individual patient. This review considers how clinical pharmacists might use such population-level information, together with the globally used updated Roussel Uclaf Causality Assessment Method (RUCAM), without overstating what either can show. We bring together evidence on culprit antibiotics and their characteristic phenotypes, the mechanistic basis of susceptibility markers including HLA-B*57:01 and slow NAT2 acetylator status, the methods and limits of disproportionality analysis, and the pharmacist’s role in medication review and monitoring. On that basis we propose a closed surveillance cycle. A pharmacovigilance signal first informs risk stratification and the timing of laboratory monitoring; suspected cases then undergo updated RUCAM assessment, which yields a quantitative causality score at no marginal cost, with genotyping reserved for patients in whom the result would change interpretation; intervention and structured reporting close the loop. Published antibiotic-associated cases are tabulated with their RUCAM scores and susceptibility results. Research priorities include prospective validation of composite risk scores, agreed monitoring intervals, multicenter evaluation of pharmacist-led detection, and appraisal of emerging biomarkers.

## 1 Introduction

Idiosyncratic drug-induced liver injury (DILI) is difficult to diagnose. The reason is simple: no single test can confirm it. The clinical manifestations, biochemical patterns, and time intervals from exposure to injury vary significantly among different patients. Therefore, diagnosis relies on ruling out other causes of liver disease and determining whether the onset time matches the suspected drug (Andrade et al., 2019; Chalasani et al., 2021). The burden of the disease in this population cannot be ignored. A prospective study in Iceland reported an annual incidence rate of 19.1 per 100,000, while a national analysis in China estimated it to be 23.8 per 100,000 (Björnsson et al., 2013; Shen et al., 2019). The prognosis also varies. In patients with hepatocellular injury accompanied by jaundice, approximately one-tenth die or require liver transplantation; this association was first described by Hy’s law and later supported by registry study data (Reuben, 2004; Andrade et al., 2005). In a significant proportion of idiosyncratic cases, a significant burden of illness and death can still be observed 6 months after onset (Fontana et al., 2014).

Antibiotics deserve particular attention, as they consistently represent the most prominent category of relevant drugs in prospective DILI cohorts. In the US Drug-Induced Liver Injury Network, antibacterial drugs account for the highest proportion of cases; in the US and European registry studies, amoxicillin-clavulanate is the most frequently identified single drug (Chalasani et al., 2008; Andrade et al., 2005; Björnsson, 2016; deLemos et al., 2016). However, “antibiotic-related liver injury” is not a unified clinical entity. Different drugs can cause different patterns of damage and latency periods, and certain associations are significantly influenced by host genetic factors. For instance, HLA-B*57:01 can increase the risk of flucloxacillin-related DILI by approximately 80 times, while NAT2 slow acetylation type is associated with liver toxicity during antituberculosis treatment (Daly et al., 2009; Yang et al., 2019). Large electronic health record cohorts have begun to quantify the specific risks of different antibiotics in routine medical treatment (Gu et al., 2023).

Spontaneous-reporting databases provide an alternative perspective. Analyses of the US Food and Drug Administration Adverse Event Reporting System (FAERS) and other databases have found particularly strong hepatotoxicity signals for amoxicillin-clavulanate and nitrofurantoin among oral antibiotics (Sebastian et al., 2026; He et al., 2025). These findings help identify drugs that warrant closer attention, but their interpretation requires restraint. Without an exposure denominator, disproportionality measures cannot provide incidence or relative risk, and reporting is affected by publicity, severity, novelty, and underreporting (Rothman et al., 2004). A further limitation is decisive for how such signals may be used: the individual reports that generate them are, as a rule, not assessed for causality by a validated scoring method, so a database signal cannot by itself establish that any of its constituent reports was truly drug-induced (Teschke and Danan, 2020). The result is an unresolved clinical problem: pharmacovigilance can identify a population-level signal, but the prescriber still has to decide what that signal means for the patient in front of them.

Clinical pharmacists are well placed to help make that decision. Their routine work already includes prescription review, therapeutic monitoring, medication reconciliation, and adverse-event reporting. In a single-center propensity-score-matched cohort, active pharmacist consultation for suspected DILI was associated with a higher 180-day recovery rate and earlier identification and withdrawal of the implicated drug (Li et al., 2022). Pharmacist-led use of the updated Roussel Uclaf Causality Assessment Method (RUCAM) has also uncovered substantial underreporting among hospitalized patients (Ortiz et al., 2025). The updated RUCAM is not a confirmatory test, but its explicit scoring rules make the reasoning behind a causality judgment visible and reviewable, and it is by a wide margin the most extensively applied instrument of its kind: a comprehensive analysis of the published literature from 1993 to mid-2020 identified 81,856 DILI and 14,029 herb-induced liver injury cases assessed by RUCAM, a body of use that underlies its description by many investigators as the field’s reference standard for causality (Danan and Teschke, 2016; Teschke and Danan, 2020).

We therefore propose a pharmacist-led approach to antibiotic-associated liver injury (AALI) that begins with a pharmacovigilance signal but does not end there. The model follows the patient from prescription review through risk stratification, laboratory surveillance, updated RUCAM-based assessment, intervention, and structured reporting back to the pharmacovigilance system (Figure 1). Previous reviews have discussed the pharmacist’s contribution to DILI care, the mechanisms and management of DILI, or antibiotic-specific reporting signals as largely separate topics (Hosack et al., 2023). Here, those elements are treated as parts of one clinical sequence. The aim is not to turn a database signal into a diagnosis. It is to use the signal, together with the expected phenotype, latency, and host factors, to decide who should be monitored, when testing should occur, and how a suspected case should be scored and documented.

**FIGURE 1:**
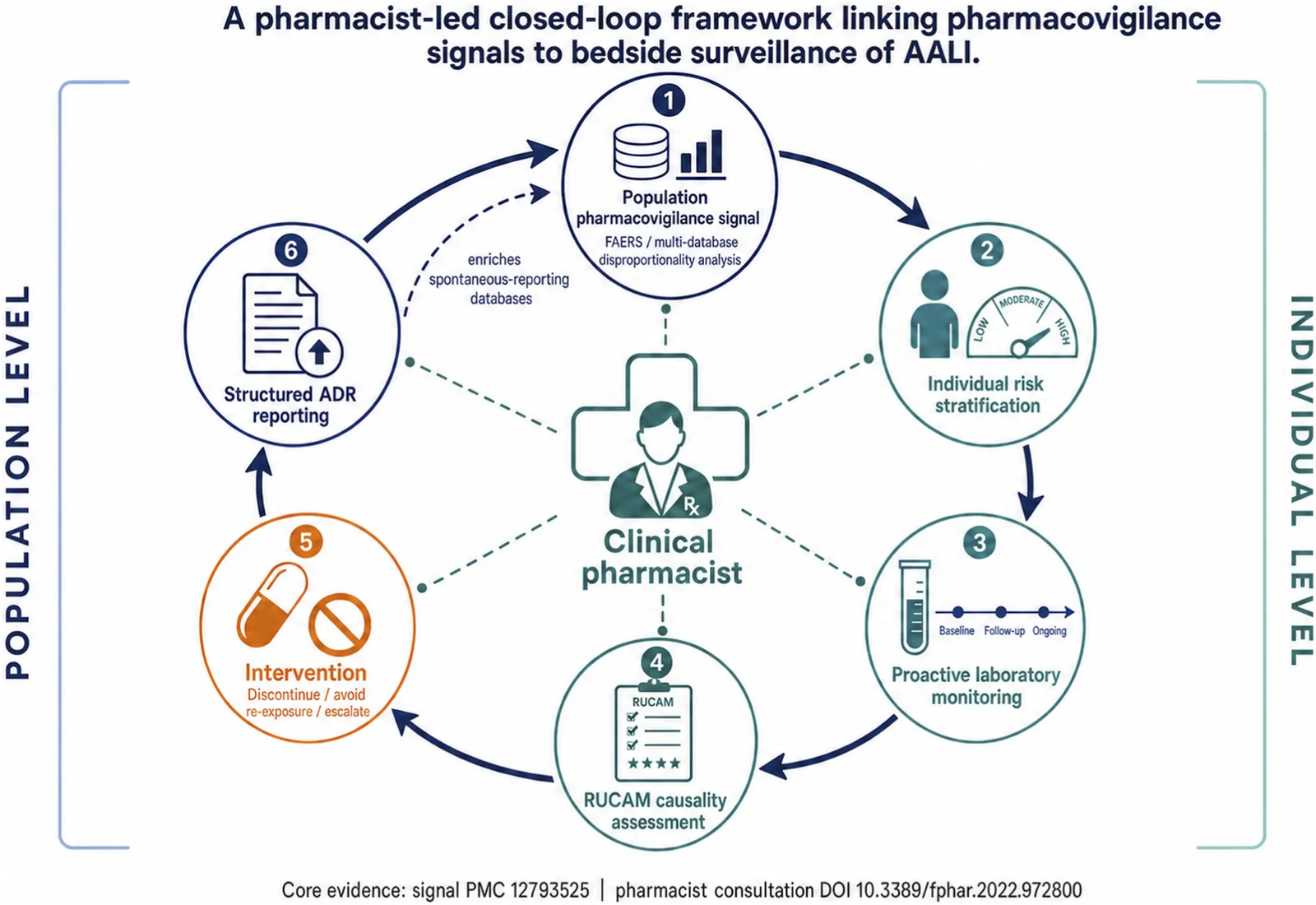
A pharmacist-led closed-loop framework linking pharmacovigilance signals to bedside surveillance of antibiotic-associated liver injury (AALI). Six sequential nodes are traversed clockwise: (1) population-level pharmacovigilance signal detection (e.g., disproportionality analysis of spontaneous-reporting databases such as FAERS); (2) individual risk stratification; (3) proactive laboratory monitoring; (4) causality assessment with the updated RUCAM; (5) intervention (withdrawal of the suspected agent, avoidance of re-exposure, and adjustment of concomitant hepatotoxins or escalation of care); and (6) structured adverse-drug-reaction reporting. A feedback arc (node 6 to node 1) returns each confirmed case, with its causality score, to the spontaneous-reporting databases that generated the original signal, making the process a closed loop rather than a linear pathway. The clinical pharmacist operates across all nodes, bridging the population level (left) and the individual patient level (right). FAERS, FDA Adverse Event Reporting System; RUCAM, Roussel Uclaf Causality Assessment Method.

## 2 The landscape of antibiotic-associated liver injury

AALI is the most common form of idiosyncratic DILI in the Western world. Yet its epidemiology, culprit list, and clinical patterns vary enough that a single monitoring rule cannot serve every agent. The drug, the mode of injury, and the latency period jointly determine when and how the injury will occur; any risk-based monitoring plan must be adapted to this heterogeneity.

Population studies on drugs themselves have not only shown the overall scale of DILI but also revealed the prominent contribution of antibiotics. In the prospective cohort in Iceland, the crude annual incidence rate was 19.1 per 100,000, and amoxicillin-clavulanate was the most common causative drug (Björnsson et al., 2013). The Chinese national study provided a higher estimate of 23.8 per 100,000, with antituberculosis drugs ranking among the top causes of Western medicine, although the case-ascertainment method used in that study has been the subject of published correspondence and its incidence estimate should be treated with corresponding caution (Shen et al., 2019). The drug registry studies reached similar conclusions: in the US drug-induced liver injury network, the proportion of cases caused by antibacterial drugs was the highest, and it also ranked first in the Spanish registry study (Chalasani et al., 2008; Andrade et al., 2005). These prospective projects recruit patients actively and adjudicate each case against competing explanations, which distinguishes them from passive report collections (Fontana et al., 2009); subsequently, the research networks in Latin America and Europe further expanded the geographical coverage of the evidence (Bessone et al., 2016; Björnsson et al., 2023). As prescription practices change, the composition of pathogenic drugs will also change, but amoxicillin-clavulanate remains one of the main causes (Fontana et al., 2023). Its typical manifestations are mixed or cholestatic damage, and it is particularly common in elderly patients (deLemos et al., 2016). Currently, electronic health record studies, including the “All of Us” research project analysis, are examining commonly used antibiotics at the population scale to complement the results of registry studies (Gu et al., 2023).

The differences in clinical patterns of various drugs can significantly affect the monitoring strategies. Flucloxacillin typically causes cholestatic-type damage, with most cases occurring within the first 6 weeks of exposure (Russmann et al., 2005). Early outbreaks of cholestatic hepatitis associated with this drug are helpful in establishing this short-latency, immune-mediated damage pattern of beta-lactam drugs (Jick et al., 1994; Devereaux et al., 1995). The course of nitrofurantoin is different. Although it can also present acutely, the more characteristic chronic type often emerges several months or even years after preventive medication and may resemble autoimmune hepatitis (Park et al., 2021; Bessone et al., 2023). Azithromycin can cause damage after the treatment period, making the exposure timeline less obvious (Martínez et al., 2015). Minocycline is also associated with a longer latent autoimmune hepatitis phenotype and significant autoantibodies (Goldstein et al., 2000; Harmon et al., 2018). Where an autoimmune presentation is suspected, recent work has argued that drug-induced autoimmune hepatitis should be diagnosed only when both components are scored separately, the drug component by the updated RUCAM and the autoimmune component by the simplified autoimmune hepatitis score, since reports applying only one of the two provide insufficient criteria for the diagnosis; the same body of work proposes a refined classification of idiosyncratic DILI subtypes on a RUCAM-ascertained basis (Teschke et al., 2025; Teschke, 2026). Fluoroquinolones and macrolides more often cause acute hepatocellular or mixed-type damage, which usually resolves after discontinuation; tetracyclines historically were associated with microvesicular fatty change, while in modern usage they often present as less severe hepatocellular damage (Andrade and Tulkens, 2011; LiverTox, 2026). These differences make phenotypes and latency periods useful diagnostic evidence, rather than just *post hoc* descriptions (Figure 2).

**FIGURE 2:**
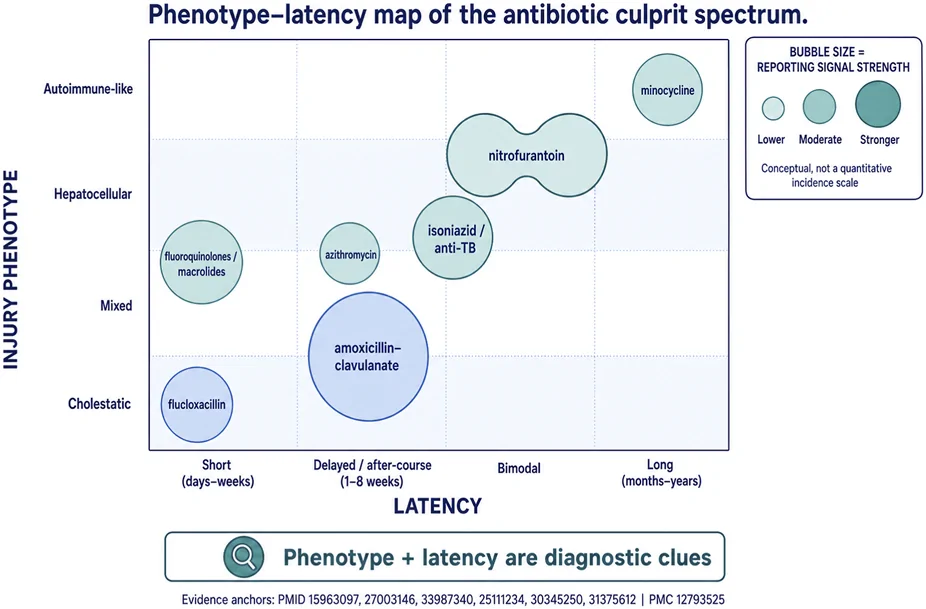
Phenotype-latency map of the antibiotic culprit spectrum. Each bubble locates a representative antibiotic (or class) by biochemical injury phenotype (y-axis: cholestatic, mixed, hepatocellular, autoimmune-like) and typical latency to onset (x-axis: short [days-weeks], delayed/after-course, bimodal, long [months-years]). Bubble size is proportional to the strength of the reporting signal. Amoxicillin-clavulanate and nitrofurantoin carry the largest bubbles, reflecting the strongest disproportionality signals. The map illustrates that phenotype and latency are themselves diagnostic clues at the bedside.

Duration of exposure is a second axis of heterogeneity, and it extends well beyond tuberculosis. Antituberculosis therapy is the familiar example, with a conventional regimen running six to 9 months, but several other indications commit patients to weeks or months of antibacterial treatment: chronic osteomyelitis, infective endocarditis, prosthetic joint infection, and other deep-seated infections managed on outpatient parenteral antimicrobial therapy (OPAT). The scale of biochemical disturbance in these settings is now quantified. In a multicenter Dutch cohort of 1,152 adults receiving OPAT, 45.1% developed a laboratory abnormality, and hepatotoxicity was the most frequent of these at 33.9 events per 1,000 OPAT days, although only 2% of patients discontinued therapy as a result; notably, the hazard of a first hepatotoxic event fell with time on treatment, which argues for front-loading rather than evenly spacing liver-function checks (Stoorvogel et al., 2024). Prolonged intravenous regimens also differ among agents that are used interchangeably in practice, so the choice of agent is itself a monitoring variable (Viehman et al., 2016). Table 1 therefore reports, for each high-risk agent, the indication and regimen that typically generate the exposure, alongside the expected phenotype, latency window, and suggested monitoring start.

**TABLE 1:** High-risk antibiotics: indication and regimen, injury phenotype, latency, and suggested monitoring.

Antibiotic	Typical indication and regimen	Typical phenotype	Latency window	Suggested monitoring start	Key susceptibility	References
Amoxicillin-clavulanate	Respiratory, ear-nose-throat, skin and urinary infections; oral, usually 5–14 days	Cholestatic/mixed	∼1–8 weeks, often after cessation	Baseline + week 2–4; review if new jaundice	Older age; repeated courses	deLemos et al. (2016)
Flucloxacillin	Staphylococcal skin, soft-tissue and bone infection; oral or intravenous, days to several weeks	Cholestatic	Short, days to a few weeks	Baseline + week 1–2	HLA-B*57:01; older age	Andrews and Daly (2008); Daly et al. (2009); Teixeira et al. (2020)
Nitrofurantoin	Acute uncomplicated urinary tract infection (5–7 days), or long-term prophylaxis (months to years)	Hepatocellular/autoimmune-like	Bimodal: Acute (days); chronic (months-years, prophylaxis)	Baseline + periodic during long-term use	Female sex; long-term prophylaxis	Park et al. (2021); Bessone et al. (2023)
Azithromycin	Respiratory and genital infections; short oral course, typically 3–5 days	Hepatocellular/mixed	Often after a short course ends	Vigilance during and shortly after course	Not established	Martínez et al. (2015)
Minocycline	Acne vulgaris and related dermatological indications; oral, months to years	Autoimmune-like hepatocellular	Long, months to years	Consider with long courses; check autoantibodies	Younger patients; prolonged therapy	Goldstein et al. (2000); Harmon et al. (2018)
Isoniazid-containing antituberculosis regimen	Pulmonary and extrapulmonary tuberculosis; isoniazid with rifampicin, pyrazinamide and ethambutol, 6–9 months	Hepatocellular	Weeks to months	Baseline + monthly per tuberculosis protocol	Slow NAT2 acetylator; CYP2E1 variants	Wang et al. (2016); Suvichapanich et al. (2018); Cavaco et al. (2022)
Prolonged parenteral regimens on OPAT (anti-staphylococcal penicillins, cephalosporins, glycopeptides)	Infective endocarditis, chronic osteomyelitis, prosthetic joint infection; intravenous, commonly 4–6 weeks or longer	Agent-dependent; hepatocellular or cholestatic	Hazard of a first hepatotoxic event is highest early and declines with time on treatment	Baseline + weekly during the opening weeks, then spaced	Concomitant hepatotoxic medication; abnormal baseline liver tests	Viehman et al. (2016); Stoorvogel et al. (2024)

OPAT, outpatient parenteral antimicrobial therapy. Suggested monitoring start times are pragmatic recommendations derived from each agent’s characteristic latency and are not validated schedules. Reporting-signal strength for these agents is discussed in Sections 2 and 4 rather than tabulated here, because a disproportionality signal is a property of a database rather than of a prescription.

The consistency of registry and population data gives pharmacists a defensible list of high-risk agents to prioritize, and the phenotype-latency signatures offer a rational basis for tailoring the timing of laboratory monitoring to each drug. Curated resources can support this prioritization provided their evidentiary basis is kept in view. The DILIrank reference list ranks more than a thousand drugs by their likelihood of causing hepatotoxicity (Chen et al., 2016), and the LiverTox knowledge base provides agent-level narrative summaries that a pharmacist can consult at the point of care (Hoofnagle et al., 2013). Both compile published and reported cases of widely varying diagnostic rigour, and neither applies a uniform validated causality assessment to the cases it aggregates; they are therefore useful for ranking attention and for orientation to an unfamiliar agent, but they cannot substitute for case-level scoring in an individual patient. No comparable resource maps culprit agent to expected phenotype, latency window, and monitoring action in a form that can be applied prospectively at the point of prescribing rather than reconstructed after injury. Table 1 assembles that mapping for the high-risk agents considered here.

## 3 Mechanisms as a source of actionable risk markers

Mechanism matters here for what it yields in the way of actionable risk markers. The immunologic and metabolic pathways that damage the liver also produce the genetic and phenotypic signals a pharmacist can act on, which is what gives mechanism its clinical purchase in a surveillance framework (Figure 3).

**FIGURE 3:**
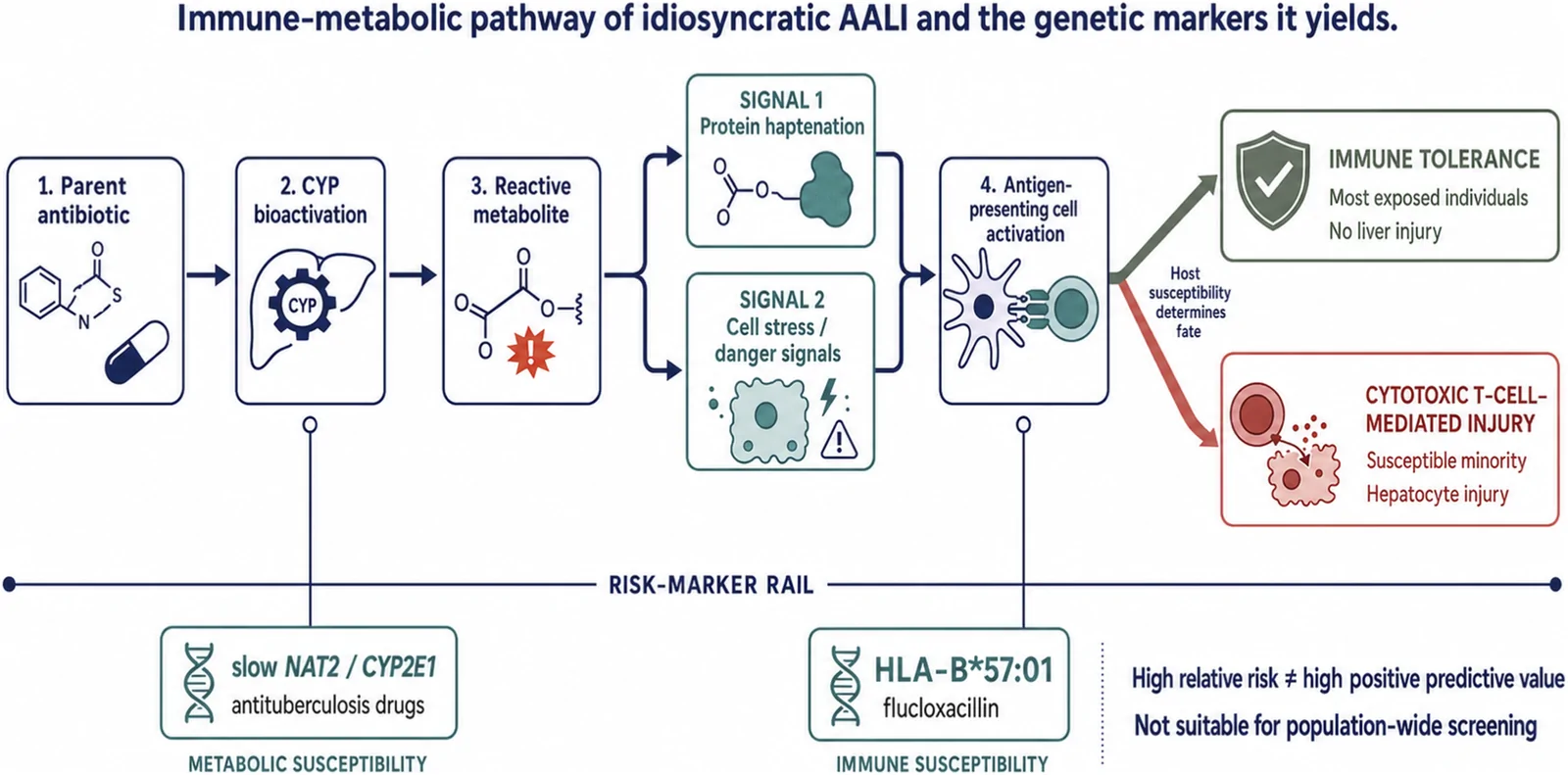
Immune-metabolic pathway of idiosyncratic AALI and the genetic markers it yields. From left to right: the parent antibiotic undergoes cytochrome P450 (CYP) bioactivation to a reactive metabolite, which haptenizes hepatic proteins (first signal) while cell stress and danger signals provide the second signal, together activating antigen-presenting cells. The immune response then bifurcates into immune tolerance (the default outcome in the majority, without clinically apparent injury) versus cytotoxic T-cell-mediated hepatocellular injury (in a susceptible minority). The lower rail anchors actionable susceptibility markers to their mechanistic step: HLA-B*57:01 (flucloxacillin) at the immune-recognition step, where drug-modified peptides are processed and presented to CD8^+^ T cells; and slow NAT2 acetylator status together with CYP2E1 variants (antituberculosis drugs) at the metabolic step, where reduced acetylation shifts isoniazid toward reactive intermediates and mitochondrial stress. These markers carry high relative risk but low positive predictive value and are therefore not recommended for population-wide screening. CYP, cytochrome P450; HLA, human leukocyte antigen; NAT2, N-acetyltransferase 2.

### 3.1 The two-signal model of idiosyncratic injury

Most idiosyncratic AALI is immune-mediated and initiated by reactive metabolites. Bioactivation of the parent drug generates reactive species that haptenize hepatic proteins, supplying the first signal for an adaptive immune response. Cell stress and danger signals provide the second (Cho and Uetrecht, 2017). This two-signal logic sits within a broader model in which an initial upstream insult, whether metabolic, oxidative, or mitochondrial, converges on shared downstream pathways of hepatocyte death (Russmann et al., 2009). Current mechanistic-clinical syntheses treat this staged process as the organizing principle for the field (Jee et al., 2021). Tolerance is the default outcome of the initial immune encounter, which explains why only a small minority of exposed patients develop injury and why many tolerate continued treatment (Uetrecht and Naisbitt, 2013). When tolerance fails, injury is largely mediated by cytotoxic T cells but depends on prior innate activation of antigen-presenting cells (Mak and Uetrecht, 2015). This immune-centered account is the one current mechanistic reviews settle on (Uetrecht, 2019).

### 3.2 Mechanism-based susceptibility markers: HLA-B*57:01 and NAT2

Two markers recur throughout this review, and their clinical standing rests on how well their biology is understood. They are worth setting out in mechanistic detail, because the reason they behave so differently at the bedside is that they act at different steps of the pathway described above.

HLA-B*57:01 acts at the immune-recognition step. Class I HLA molecules present short peptides on the hepatocyte surface for inspection by CD8^+^ cytotoxic T cells. Flucloxacillin, or a reactive metabolite generated from it, binds covalently to hepatic and serum proteins, and the resulting drug-modified peptides are processed and loaded onto HLA-B*57:01; mass-spectrometric work has directly identified flucloxacillin-haptenated HLA-B*57:01 ligands, confirming that the modified peptide is genuinely processed and presented rather than merely bound at the cell surface (Waddington et al., 2020). Naive T cells from HLA-B*57:01-positive donors are activated by flucloxacillin when the drug antigen is presented by dendritic cells, and the responding clones proliferate and secrete interferon-gamma, which supplies the immunological basis for the clinical association (Monshi et al., 2013). Genotype carries a correspondingly large relative effect: carriers have an approximately 80-fold higher risk of flucloxacillin DILI (Daly et al., 2009). The phenotype may also reflect more than one pathway. CYP3A4/3A7-mediated bioactivation can account for cholestatic injury, whereas development of a hepatocellular pattern appears to require additional immune activation (Andrews and Daly, 2008; Teschke, 2024). Series that combine HLA typing with causality assessment by the updated RUCAM are consistent with this metabolic-to-immune progression (Teschke and Danan, 2024).

NAT2 acts one step earlier, on drug disposition. N-acetyltransferase 2 is the phase II enzyme that acetylates isoniazid, and slow acetylator genotypes reduce that clearance route, shifting more of the dose through alternative pathways that yield hydrazine and other reactive intermediates; these species have been linked experimentally to mitochondrial oxidant stress and impaired energy homeostasis, with underlying complex I impairment able to convert otherwise tolerated isoniazid concentrations into severe hepatocellular injury (Boelsterli and Lee, 2014). This account fits the clinical picture of antituberculosis DILI, which is typically hepatocellular and emerges over weeks to months of continuous exposure rather than within days. It should be stated plainly, however, that the mechanism is not settled: the same analysis notes that the contribution of NAT2 and CYP2E1 polymorphisms has become less prominent as HLA associations have been recognized in isoniazid injury, and that genotype-phenotype correlations of this kind remain correlative. The epidemiological association is nonetheless reproducible. Slow acetylators are at higher risk and ultra-slow acetylators highest (Suvichapanich et al., 2018; Yang et al., 2019), multiple meta-analyses confirm the association (Wang et al., 2012; Khan et al., 2019), CYP2E1 appears to add a smaller effect (Wang et al., 2016), and combinations of metabolic-enzyme genotypes may separate susceptibility better than any single locus (Cai et al., 2012). In a Portuguese cohort of 233 patients treated with a standard antituberculosis protocol, in which all mild cases scored at least 4 and all DILI cases at least 6 on the updated RUCAM, NAT2 slow acetylator status was an independent risk factor alongside age of 55 years or more, concomitant hepatotoxic medication, and an ABCB11 variant, though the full model explained only about a quarter of susceptibility (Cavaco et al., 2022).

### 3.3 Why these markers inform rather than decide

These markers have informational value, but they cannot yet be used independently as screening tools. HLA-B*57:01 illustrates the issue: although its relative effect is large, flucloxacillin DILI is rare even among carriers, so the positive predictive value of a positive test remains very low (Daly et al., 2009). Routine population-wide typing before a common antibiotic is therefore difficult to justify on present evidence. Using NAT2 genotype to guide isoniazid dosing is biologically reasonable and may improve the safety of antituberculosis treatment, but it is not yet standard care, and the available health-economic evaluation comes from a single national setting rather than from multinational data (Kantared and Permsuwan, 2026). Two practical consequences follow, and they set up the sequence described in Section 5.3. First, a genotype that is already on file should shift an assessment rather than settle it. Second, because these tests carry real cost while causality assessment does not, susceptibility testing belongs after, not before, a structured causality judgment. The more useful question in current practice is how to combine an available genotype with the patient’s age, comorbidities, concomitant medications, expected antibiotic exposure, and the strength of the pharmacovigilance signal. Such a comprehensive approach still requires prospective validation.

## 4 Real-world pharmacovigilance signals and their interpretive limits

There are no patients in the spontaneous reporting database; only reports. This fact determines what the analysis results can express. Among millions of records, disproportionate analysis can uncover any drug-event combinations that individual clinicians would find difficult to detect, but it cannot provide the number of patients who took the drug but did not suffer any harm. For antibiotic hepatotoxicity, the analysis yields report signals rather than measured probabilities of injury occurrence; any subsequent clinical use must inherit and address this limitation.

The underlying computation is a two-by-two comparison. Take the reports that name one antibiotic, count how many of them also mention liver injury, and set that fraction against the same fraction computed across every other drug in the database. The proportional reporting ratio and the reporting odds ratio differ mainly in how they form that quotient. The information component instead pulls unstable estimates toward the database average through a Bayesian confidence propagation neural network (Bate et al., 1998; Evans et al., 2001; van Puijenbroek et al., 2002). None of the three is definitive. Head-to-head comparisons of the reporting odds ratio and the proportional reporting ratio have not established a universally superior method, which is why contemporary studies tend to report several statistics side by side and sometimes add the empirical Bayes geometric mean as a further check (Rothman et al., 2004; Waller et al., 2004).

When applied to antibiotics, the resulting ranking was generally stable. Among commonly used oral agents, amoxicillin-clavulanate and nitrofurantoin are both among the drugs with the strongest hepatotoxicity signals in FAERS, although the biochemical patterns reported for the two are not the same (Sebastian et al., 2026). Studies spanning several adverse-reaction databases support these associations and add time-to-onset distributions, in which many events are reported within weeks of starting the drug (He et al., 2025). Classes that attract less attention have been examined the same way, carbapenems among them (Tang et al., 2025).

Multiple biases can act simultaneously on the original reported numbers. Firstly, the system does not account for the denominator: it records the received reports rather than the actual patients who received the medication, so the reporting odds ratio cannot be interpreted as a relative risk. Secondly, the reports are selective, with serious, novel or media-covered events being more likely to be submitted, while familiar reactions are often not reported. Underreporting is very common, and the reporting bias also changes over time, so an increased signal may reflect an increase in attention rather than an increase in the incidence rate (Rothman et al., 2004). Thirdly, and most consequentially for the framework proposed here, the constituent reports are heterogeneous in diagnostic quality and are typically submitted without any validated causality assessment, so the cases behind a signal have not been shown individually to be drug-induced (Teschke and Danan, 2020). What follows in practice is that a signal is a prompt to investigate, and its credibility is judged by what else agrees with it: population-based incidence data from prospective cohorts with adjudicated cases, a plausible mechanism, the phenotype one would expect of that drug, and, with the caveats set out in Section 2, curated classifications such as DILIrank and LiverTox (Chen et al., 2016; Hoofnagle et al., 2013). Convergence across those sources makes a signal more useful clinically. It still does not establish causality, in the database or in the patient.

At the bedside the signal does two things. It sets how closely a patient is watched, and it shortens the interval before a particular antibiotic is suspected once liver tests move in the wrong direction. It also gives stewardship a defensible order of priority. What it cannot do is name the culprit in an individual case. That judgment still rests on the exposure timeline, competing causes, the response to dechallenge, the biochemical pattern, and, where the question arises, rechallenge, which are precisely the domains the updated RUCAM scores. In the workflow proposed here, pharmacovigilance sets the initial level of suspicion and patient-level evidence tests it.

## 5 From signals to bedside: Pharmacist-led active surveillance

The proposed workflow gives the clinical pharmacist a defined role before, during, and after antibiotic exposure. It begins by deciding which patients require more than routine observation. Monitoring is then timed to the expected latency of the prescribed drug, and any suspected case is assessed using a structured causality method. These steps are related, but each answers a different question: who is vulnerable, when injury is most likely to appear, and whether the antibiotic is a credible cause.

### 5.1 Risk stratification: Fusing signal, phenotype, and host susceptibility

Three questions are answerable at the moment a prescription is reviewed. How strong and how consistent is the reporting signal for this antibiotic? What injury would this drug be expected to produce, and on what timescale? And what about this patient raises or lowers susceptibility, counting age, coexisting liver disease, alcohol use, other hepatotoxic medicines already on the chart, and any genetic result that happens to be on file? Each question draws on a distinct body of evidence: disproportionality analyses for the first, agent-specific phenotype and latency data for the second, and cohort studies of host risk factors for the third, in which age of 55 years or more, concomitant hepatotoxic medication, and slow NAT2 acetylator status have all been identified as independent contributors (Sebastian et al., 2026; Russmann et al., 2005; deLemos et al., 2016; Daly et al., 2009; Yang et al., 2019; Cavaco et al., 2022). HLA-B*57:01 carriage and slow NAT2 acetylator status belong in the third category, though their low positive predictive value keeps them out of use as population screening tests. A genotype that is already available should shift an assessment rather than settle it.

All these pieces of information fall within the scope of the routine drug use review and are inherently data that would be covered. The current guidelines require clinicians to consider host and drug factors such as age, gender, alcohol consumption, daily dose, and lipophilicity (Chalasani et al., 2021). The assessment results are intentionally kept in broad stratification because there is currently no validated tool that can convert these inputs into specific probabilities; the goal is simply to classify patients into two categories: those who should be scheduled for biochemical monitoring, and those who can be given symptom education and routine follow-ups. Figure 3 and Table 1 present this comprehensive assessment, which includes both established risk factors and emerging biomarker schemes (Danjuma et al., 2019).

A worked case can make this stratification more intuitive. A geriatric inpatient started taking flucloxacillin orally and was already taking statin drugs. Flucloxacillin has a clear cholestatic injury phenotype and a short latency period, and the patient’s age and another potential hepatotoxic drug further increase the risk. If the HLA-B*57:01 test result is available, it should also be included in the judgment; but whether to routinely perform this test for each such patient is another issue that requires independent argumentation. For this case, it is a reasonable approach to conduct a liver function test before treatment and plan to recheck within the first 2 weeks. If it were a young outpatient patient who used only a weakly signaling antibiotic for a short period and had no other risk factors, then providing symptom warnings and routine clinical follow-ups would be sufficient, commensurate with the risk. Table 1 applies the same reasoning to the various high-risk antibiotics discussed in this article.

### 5.2 Proactive laboratory monitoring

The monitoring plan should be formulated based on the typical ways in which each drug causes damage. Drugs with short latency periods, such as flucloxacillin, should be monitored earlier; while for long-term use of nitrofurantoin for prevention, the alert period should be much longer than the initial few weeks (Russmann et al., 2005; Park et al., 2021). For prolonged parenteral regimens the empirical hazard pattern points the same way, with the risk of a first hepatotoxic event highest early in the course and declining thereafter, which supports concentrating tests in the opening weeks rather than testing at a fixed interval throughout (Stoorvogel et al., 2024). Whether to escalate should also be based on the biochemical damage pattern and the clinical severity.

The hepatocellular type of injury accompanied by jaundice conforms to the core situation emphasized by Hy’s law, and is associated with a risk of approximately 10% of death or liver transplantation (Reuben, 2004; Temple, 2006). How that rule should be operationalized has been refined considerably since it was first codified. The algorithm combining the R ratio with bilirubin and albumin was proposed to enhance the predictive ability for acute liver failure and to provide a clearer threshold for emergency assessment (Robles-Díaz et al., 2014). More recent work addresses the situations in which the classical definition performs least well, namely, abnormal baseline liver tests, cholestatic phenotypes, and newer drug classes, and proposes revised criteria for identifying hepatocellular injury when alkaline phosphatase is also raised (Cohen and Lewis, 2025). In a regulatory case series, a normalized R value of 5 or more identified Hy’s law cases with substantially greater sensitivity for fatality and acute liver failure than the conventional requirement that alkaline phosphatase be less than twice the upper limit of normal, although the number of severe outcomes was small and positive predictive values were low for both criteria (Amirzadegan et al., 2026). For a pharmacist, the practical implication is that the R ratio should be calculated and re-calculated as the injury evolves rather than applied once at presentation. The decision to discontinue medication should follow established biochemical standards and be combined with the patient’s clinical context (Andrade et al., 2019; Chalasani et al., 2021). In regions where antituberculosis drugs and traditional drugs account for a large proportion of exposure, local guidelines should also be considered (Mao et al., 2024).

Structured diagnostic and prognostic pathways for suspected DILI translate these thresholds into an ordered sequence of bedside decisions (Andrade and Robles-Díaz, 2020). Figure 4 maps this monitoring logic onto the decision points of the surveillance workflow.

**FIGURE 4:**
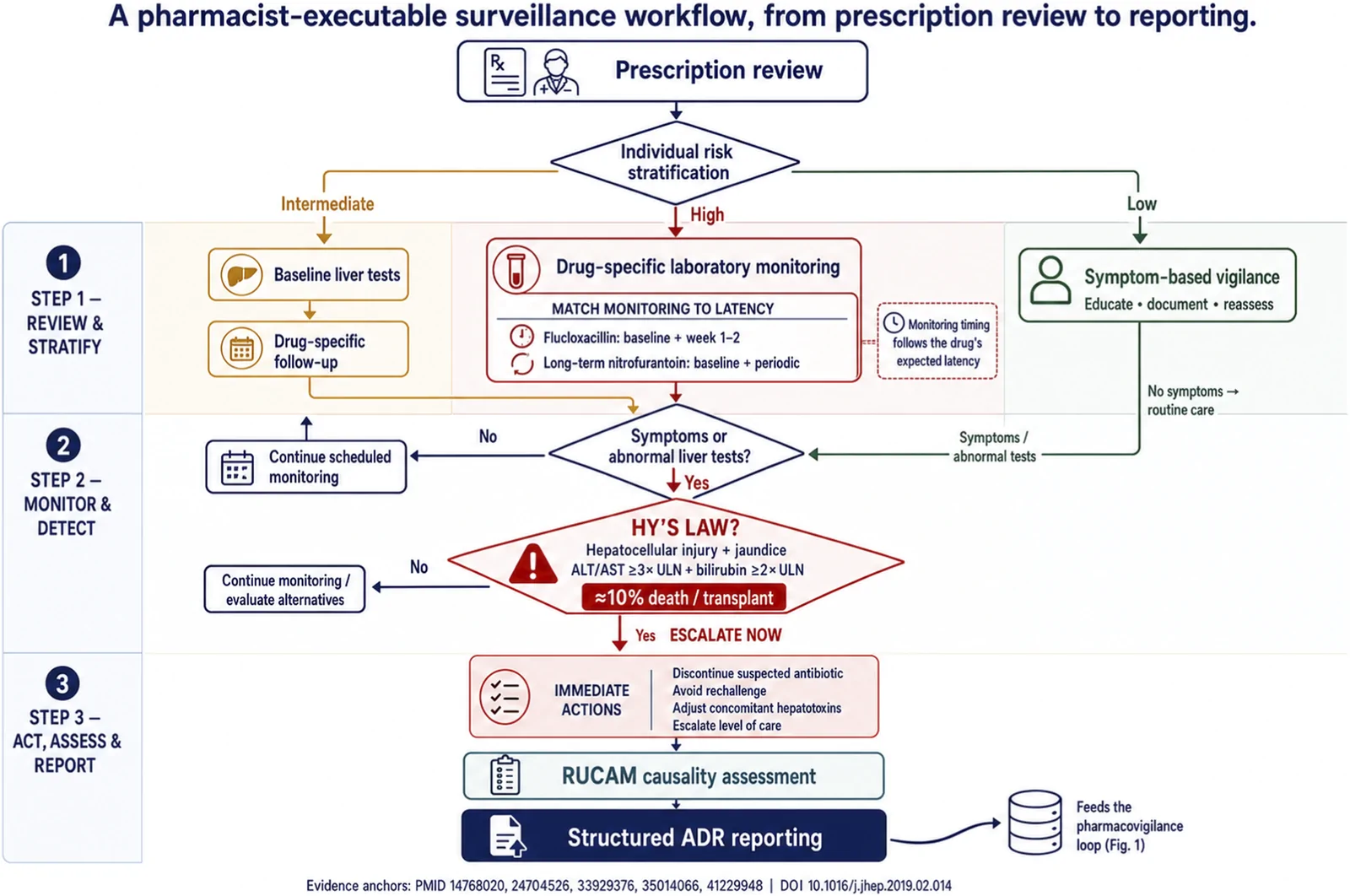
A pharmacist-executable bedside surveillance workflow, from prescription review to reporting. The swim-lane flow begins with prescription review and risk stratification (high/intermediate/low). Drug-specific monitoring time points follow (for example, baseline plus weeks 1–2 for flucloxacillin; baseline plus periodic testing during long-term nitrofurantoin prophylaxis). At the interpretation node, Hy’s law (hepatocellular injury with jaundice, associated with roughly 10% risk of death or transplantation) triggers escalation. Actions comprise discontinuation, avoidance of re-challenge, adjustment of concomitant hepatotoxins, and escalation of care, followed by scoring with the updated RUCAM and structured reporting; low-risk cases follow a symptom-based vigilance branch. Monitoring intensity is matched to each drug’s characteristic latency, and the endpoint returns to reporting, feeding the loop shown in Figure 1. RUCAM, Roussel Uclaf Causality Assessment Method.

### 5.3 Structured causality assessment with the updated RUCAM

Structured causality assessment closes the diagnostic step and is where pharmacist involvement adds measurable value. The updated RUCAM provides a transparent, quantitative score that suits routine pharmacist use, with explicit domains for temporal sequence, dechallenge, competing causes, concomitant drugs, risk factors, previous reports, and rechallenge (Danan and Teschke, 2016; Danan and Teschke, 2019). It has been applied at a scale no competing instrument approaches: a comprehensive analysis of publications from 1993 to mid-2020 assembled 81,856 DILI and 14,029 herb-induced liver injury cases assessed by RUCAM, following an earlier analysis of 46,266 DILI cases published from 2014 to early 2019, and it is on the strength of this accumulated worldwide use that many investigators regard the method as the reference standard for causality in DILI (Teschke, 2019; Teschke and Danan, 2020). When pharmacists apply the updated RUCAM prospectively, they identify suspect drugs and exposures that would otherwise be missed (Ortiz et al., 2025). Structured application in hospitalized cohorts has shown that a meaningful proportion of DILI events are potentially avoidable when a pharmacist reviews the medication list against the injury pattern (Danjuma et al., 2020). A revised electronic version has been proposed with the aim of improving objectivity and inter-rater agreement (Hayashi et al., 2022); it has not accumulated a comparable record of independent application, and the updated RUCAM remains the instrument of choice for the workflow described here.

The two assessments in this framework are deliberately ordered, and the order is driven as much by cost as by logic. Causality assessment comes first and applies to every suspected case: the updated RUCAM requires only data already in the record, namely, the exposure timeline, serial liver biochemistry, the results of the exclusion work-up, and the concomitant medication list, and it therefore carries no marginal laboratory cost. Susceptibility testing comes second and applies selectively, to patients in whom a genotype would change management or interpretation. It is not free. In the United States, academic immunogenetics laboratories publish list prices for HLA-B*57:01 typing (CPT 81381) of approximately US$350 to US$375, with Medicare Clinical Laboratory Fee Schedule reimbursement substantially lower and patient liability dependent on insurance (UCLA Immunogenetics Center, 2022; UCSF Immunogenetics and Transplantation Laboratory, 2020). NAT2 genotyping is not offered as a routine billable clinical test by the major United States reference laboratories, so no comparable domestic price can be quoted; the available health-economic evidence for NAT2-stratified isoniazid dosing comes from a single-country cost-utility analysis (Kantared and Permsuwan, 2026). Because a positive genotype has low positive predictive value while a structured causality score is free and universally applicable, testing everyone before scoring anyone would invert the cost-effectiveness of the pathway. Whether the sequence proposed here is itself cost-effective has not been evaluated and should be.

Published cases allow the two steps to be inspected together, which is the question of whether susceptibility results corroborate the causality score. Table 2 assembles antibiotic-associated cases in which an updated RUCAM score was reported, together with whatever susceptibility testing was performed. The pattern is instructive. In most published reports no genotyping is done at all, and the RUCAM score carries the diagnosis unaided. Where genotyping is performed, it may agree with the causality assessment, as in a flucloxacillin case with a positive HLA-B*57:01 result, or it may fail to corroborate through the expected route, as in an amoxicillin-clavulanate case that scored 6 on the updated RUCAM but carried none of the known amoxicillin-clavulanate risk alleles. At cohort level the two lines of evidence do converge: in the Portuguese antituberculosis series, every mild case scored at least 4 and every DILI case at least 6, and NAT2 slow acetylator status was an independent predictor within that RUCAM-defined population (Cavaco et al., 2022). Taken together these observations support the ordering above. A causality score is obtainable in every case and is what makes a report interpretable, whereas a susceptibility result is obtainable in some cases and is informative when positive but not exculpatory when negative.

**TABLE 2:** Published antibiotic-associated liver injury cases assessed with the updated RUCAM, and whether susceptibility testing corroborated the causality score.

Antibiotic	Report and clinical setting	RUCAM score (category)	Susceptibility parameter and result	Did susceptibility corroborate the causality assessment?	References
Flucloxacillin	Single case; 74-year-old man treated for erysipelas on two prior occasions; cholestatic injury progressing to a chronic course	Not reported by the authors	HLA-B*57:01 screening positive	Genotype is concordant with the established association, but no causality score was published, so the two lines of evidence cannot be compared formally	Teixeira et al. (2020)
Amoxicillin-clavulanate	Single case; middle-aged woman with accompanying autoimmune features (antinuclear antibodies positive, raised IgG)	6 (probable)	HLA-targeted sequencing found none of the known amoxicillin-clavulanate risk alleles; Sanger sequencing identified ERAP2 rs1363907	Not corroborated through the expected HLA route; the causality score carried the diagnosis, and a candidate locus outside the usual panel was proposed	Zhan et al. (2025)
Nitrofurantoin	Single case; acute injury after four doses, with a disproportionate AST rise and rapid complete recovery after withdrawal	6 (probable), updated RUCAM	Not performed	No susceptibility data; causality rests on the score alone	Sampat (2026)
Meropenem	Single case; 80-year-old woman treated for catheter-associated urinary tract infection; cholestatic pattern (R value 1) on day 6	8 (probable), updated RUCAM	Not performed	No susceptibility data; causality rests on the score alone	Shrestha et al. (2026)
Piperacillin-tazobactam	Single case; hospitalized patient on an extended course; marked transaminase rise with hepatic steatosis on computed tomography	7 (probable)	Not performed	No susceptibility data; causality rests on the score alone	Maheshwari et al. (2025)
Isoniazid-containing antituberculosis regimen	Cohort of 233 patients on a standard protocol; 37 with mild hepatitis and 66 with DILI	All mild cases scored 4 or more; all DILI cases scored 6 or more	NAT2 slow acetylator status (OR 2.46), ABCB11 p.Val444Ala (OR 2.06), age 55 years or more (OR 3.67), concomitant hepatotoxic medication (OR 2.54)	Corroborated at cohort level within a RUCAM-defined population, although the model accounted for only about a quarter of susceptibility	Cavaco et al. (2022)

All entries are drawn from the published literature. This review reports no primary patient data of its own and contains no simulated or hypothetical case. AST, aspartate aminotransferase; DILI, drug-induced liver injury; OR, odds ratio; RUCAM, Roussel Uclaf Causality Assessment Method.

Applied together, these three steps should shorten the interval from exposure to recognition. Whether they do remains to be shown. Composite risk scores have not been validated prospectively, monitoring intervals for individual antibiotics have not been agreed, and it has not been confirmed that earlier pharmacist-led recognition changes hard clinical outcomes.

## 6 Closing the loop: Intervention and reporting

Detection is clinically meaningful only when it triggers action and feeds back into the systems that generated the original signal. Intervention and reporting are the steps that convert a diagnosis into improved outcomes and better future surveillance, and they are what make the pharmacist-led model a closed loop rather than a linear pathway.

Pharmacist intervention is linked to an improved course of established injury, although the direct evidence is still limited. In a single-center, propensity-score-matched cohort of inpatients with DILI, pharmacist active consultation was linked to an approximately ten-percent higher 180-day recovery rate. In the matched analysis, it was also linked to a greater likelihood of identifying the suspect drug and achieving timely withdrawal than usual care (Li et al., 2022). Because this evidence is observational, single-center, and not specific to antibiotics, it should be read as supportive rather than confirmatory. The core interventions are prompt discontinuation of the offending antibiotic, avoidance of rechallenge, adjustment of concomitant hepatotoxic therapy, and escalation of severe cases for specialist and transplant assessment consistent with guideline management (Chalasani et al., 2021). For particular high-risk regimens, pharmacist-led risk mitigation can begin before injury. In antituberculosis therapy, population data on prophylactic hepatoprotectant use illustrate how a pharmacy service can operationalize prevention as well as response (Chen et al., 2022). Contemporary management syntheses place these actions within a broader medication-safety strategy that pharmacists are equipped to lead (Tiwari et al., 2025). These actions are within the established scope of clinical pharmacy and require no new authority to implement.

Structured reporting is the step that regenerates the population signal. Pharmacists are well positioned to detect and report adverse events yet historically contribute a small share of reports. Dedicated pharmacist programs measurably increase both the number and quality of adverse-drug-reaction reports. Embedding clinical pharmacy residents in the reporting workflow raises report volume (Baniasadi et al., 2014). A multiyear interrupted time-series analysis shows that a sustained pharmacist intervention produces a durable increase in reporting rather than a transient spike (Lan et al., 2022). The study evidence for pharmacist-led detection and reporting of DILI is summarized in Table 3. Quality matters more than volume here, and it is the point at which this framework repays the databases it draws on. A report that carries an updated RUCAM score, the domain-level reasoning behind it, the biochemical pattern, and the latency is a qualitatively different object from a bare drug-event pair, because it can be re-assessed by someone else. If a meaningful share of reports arrived in that form, the case series behind a disproportionality signal would no longer be of unknown diagnostic quality, which is the central limitation identified in Section 4. This feedback is what distinguishes a closed loop from one-directional care and links individual practice to system-level pharmacovigilance.

**TABLE 3:** Pharmacist-led detection and reporting of drug-induced liver injury: study evidence.

Study	Design	Population	Pharmacist intervention	Main outcome	Evidence level
Li et al. (2022)	Propensity-score-matched cohort (single-center)	Inpatients with DILI	Pharmacist active consultation	Higher 180-day recovery; earlier withdrawal of the suspected drug	Observational (moderate)
Ortiz et al. (2025)	Pharmacist-led prospective assessment	Hospitalized patients	Prospective RUCAM application	Identified missed suspect drugs; exposed underreporting	Observational
Baniasadi et al. (2014)	Before/after	ADR reporting program	Clinical pharmacy residents in reporting	Increased ADR report volume	Observational
Lan et al. (2022)	Interrupted time series (9-year)	Hospital-wide ADR reporting	Sustained pharmacist intervention	Durable increase in reporting rate	Quasi-experimental
Dighriri et al. (2023)	Systematic review	Antimicrobial stewardship	Pharmacist-led stewardship	Improved prescribing and safety outcomes	Systematic review

ADR, adverse drug reaction; DILI, drug-induced liver injury; RUCAM, Roussel Uclaf Causality Assessment Method.

Closing the loop holds out a plausible but still-hypothetical self-reinforcing cycle in which better bedside recognition produces better population data, which could in turn sharpen future risk stratification. The gaps here are practical rather than conceptual. Reporting remains incomplete, the outcome benefit of pharmacist reporting on downstream signal quality has not been quantified, and integration of case capture into routine workflow is uneven. Addressing these is less a matter of new science than of implementation.

## 7 Implementation barriers and facilitators

The framework will be useful only if it fits the way antibiotics are prescribed and monitored in practice. Most of the obstacles are not failures of pharmacology. They arise from staffing, decision-support design, professional authority, and the uneven capacity of hospitals to collect and act on laboratory data.

Electronic clinical decision support could extend surveillance beyond patients seen directly by a specialist pharmacist. An alert might identify a new rise in liver enzymes, a high-risk antibiotic, or a combination of host and drug factors. Specificity is critical, however. In one evaluation, only about half of alerts for hepatic dysfunction during hepatotoxic therapy were clinically relevant, a performance level likely to produce alert fatigue (Nguyen et al., 2025). Evidence from dose-adjustment systems in oncology suggests that prescribing can change when the alert logic is narrow and clinically interpretable (Rolvink et al., 2022). Studies of abnormal liver-function tests in primary care and electronic interventions for chronic liver disease likewise show variable effects rather than an automatic benefit from digitization (Donnan et al., 2007; Ge et al., 2025). Automated detection is therefore best used to direct pharmacist attention, not to replace clinical review.

Antimicrobial stewardship programs provide a practical setting for this work. In such programs, pharmacists have been involved in antibiotic selection, treatment course review, dose optimization, and toxicity monitoring (Parente and Morton, 2018). Incorporating liver toxicity monitoring into the existing structure might be more feasible than establishing an independent service. Systematic evidence indicates that pharmacist-led antimicrobial drug management can improve prescription and medication safety, which supports the aforementioned organizational approach, but it cannot alone prove that it can improve the outcome of DILI (Dighriri et al., 2023).

The implementation methods vary among different hospitals. In some medical systems, pharmacists can offer suggestions but cannot directly adjust the treatment. Training in causality assessment is uneven, and scoring is more demanding in some clinical situations than others. The updated RUCAM is at its most decisive when a single agent is temporally implicated and the exclusion work-up is complete; where several potentially hepatotoxic drugs are given concurrently, the instrument’s own concomitant-drug domain reduces the score attainable by each candidate agent, so attribution becomes correspondingly harder and a low score in that setting should prompt specialist review rather than reassurance (Danan and Teschke, 2016). Hospitals lacking integration of prescription records and laboratory records may be unable to detect cases automatically at all. The same limitations can also result in incomplete reporting of adverse events and make the application of composite risk scores difficult to be consistent.

Therefore, a functioning project requires a small number of highly specific triggering conditions, clear inclusion in the antimicrobial drug management system, the ability to promptly obtain liver function test results, and targeted training on phenotypic identification and causality assessment. The report should be integrated into the review process rather than being appended as an additional administrative task. There are still two unresolved issues: whether the required resources are cost-effective, and whether earlier intervention by pharmacists can improve the truly important outcomes for patients. Both need to be answered through prospective studies.

## 8 Conclusion and future directions

There is no single monitoring rule applicable to all cases of antibiotic-associated liver injury. Flucloxacillin often shows problems within a few weeks, while nitrofurantoin may remain undetected for several years. The susceptibility of different patients varies, and pharmacovigilance signals reflect reporting behaviour rather than the actual risk of any individual. A framework that is useful clinically must first hold these three kinds of information apart and read each on its own terms, then bring them together at the points where decisions are actually made, at prescribing and at follow-up.

The model proposed in this article assigns specific tasks to each type of information: the reporting signal is used to identify antibiotics worthy of attention; the phenotype and latency determine when to conduct observations; host factors determine the intensity of observations. Once changes occur in liver function tests, the updated RUCAM provides a transparent and quantitative assessment framework, applicable at no marginal cost to every suspected case, with susceptibility testing reserved for the smaller number of patients in whom a genotype would change interpretation; intervention and detailed reporting then close the loop. Based on the existing evidence, pharmacist consultations may be related to earlier identification of responsible drugs, faster discontinuation of medication, and better recovery. However, this judgment is mainly based on single-matched cohort studies and should not be mistaken as the efficacy having been proven.

Some parts of this model can now be subjected to practical tests. The composite risk score requires prospective validation; direct comparisons need to be made regarding the monitoring intervals for different antibiotics and patient groups. The gap in conclusive clinical evidence remains unfilled: there is no multicenter randomized trial to prove that pharmacist-led identification can improve severe DILI outcomes, shorten hospital stays, reduce mortality rates, or decrease liver transplants. The implementation study, in addition to reporting clinical endpoints, also needs to report the burden of alerts, compliance with the recommended testing schedule, reporting quality, and costs. The cost-sensitive sequence set out in Section 5.3, in which structured causality assessment precedes selective genotyping, is itself a testable proposition and has not been formally evaluated.

Biological markers may enable earlier identification of damage in the future. In human studies, microRNA-122 and keratin-18 can be detected earlier than the elevation of traditional aminotransferases (Thulin et al., 2014). In a large international collaborative study, keratin-18, caspase-cleaved keratin-18, and glutamate dehydrogenase demonstrated prognostic discriminatory ability, but their positioning in routine diagnosis and treatment remains undetermined (Church et al., 2019; Atallah et al., 2021; Humphries and Dear, 2023). MicroRNA-122 has been studied in cholestatic and hepatocellular damage; even under controlled treatment exposure conditions, keratin-18 can reflect changes in aminotransferases (Huang et al., 2013; Scullion et al., 2024). In a prospective cohort of antituberculosis treatment, these markers change with the treatment process rather than remaining stable (Rupprechter et al., 2021).

In large electronic health record systems, the use of machine learning to identify cases may be helpful, but its value depends on specificity and clinical integration rather than the complexity of the model. Verified risk scores, biomarkers, and monitoring tools will strengthen the first half of the cycle. More acute bedside identification will subsequently produce more complete and fully characterized reports, each carrying an updated RUCAM score that makes it re-assessable, thereby improving the drug safety evidence passed on to the next patient. This feedback is precisely the main value of this framework. Proving that it can indeed change patient outcomes is the remaining task to be accomplished.
